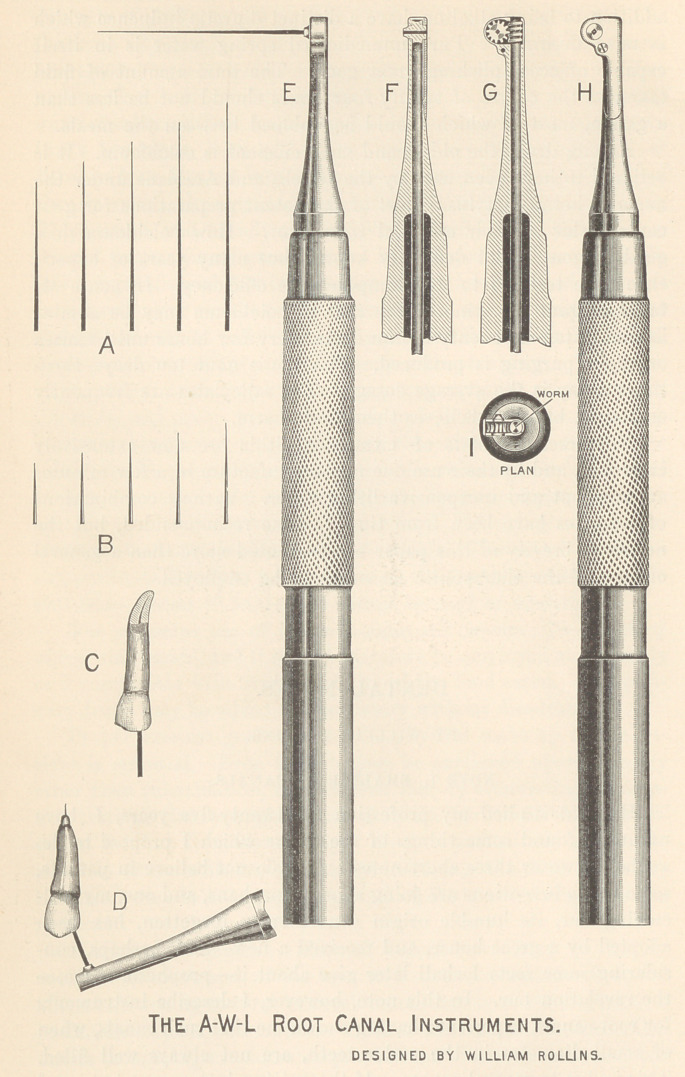# Dental Notes

**Published:** 1899-07

**Authors:** William Rollins


### DENTAL NOTES.

BY WILLIAM ROLLINS.

### NOTE I. SMALL ROOT-CANALS.

Having studied my profession for twenty-five years, I have naturally found some things of use to me which I propose to describe in two or three short notes. As I do not believe in patents, some of my inventions are going about as orphans, and one, my end-cutting bur, its humble origin conveniently forgotten, has been adopted by a great house, and received a new—and, perhaps, considering some facts I shall later give about it—prophetic name,—the revelation bur. In this note, however, I describe instruments for root-canals, experience having shown me that such canals, when of small diameter in the molar teeth, are not always well filled. This is due to several causes. If the cutting instrument is turned

THE A-W-L ROOT CANAL INSTRUMENTS.

DESIGNED BY WILLIAM ROLLINS.

by hand, it is difficult to secure room for efficient use. If used in the engine, there is the same difficulty, on account of the large size of the hand-piece which obstructs the view, and takes up most of the room which should be devoted to the instrument. In addition, the high speed, about twenty-five hundred to three thousand revolutions a minute, is almost sure to break the instrument when it is of small diameter.

I have tried to overcome some of the difficulties by means of the instrument shown in the plate. The hand-piece is arranged to attach by a sliding joint to some form of the Bonwill wrist-joint, which is the only satisfactory connecting link between the motive power and the revolving instrument used in dentistry. The object of the construction shown is to make a small instrument which will not occupy valuable space in the mouth and will run at a slow speed. The worm-gear naturally adapts itself to these conditions, so I have used it for the first time in a dental hand-piece. It is usually possible to run a dental engine as slowly as half speed, about twelve hundred revolutions a minute. This enables the pulp-canal hand-piece point to turn only one hundred times a minute, at which speed a fine instrument, properly made, should not break, if it is new.

The cutting tools are worth a short description. They are made of mild steel without being tempered by fire. When I began to use this kind of an instrument, it was not known that an untempered instrument would cut dentine satisfactorily, but in trying to overcome the constant breaking of the usual instruments in the canals, I found that if a suitable steel were drawn and hammered properly, it became hard enough and tough beyond belief. The power of a hammer is marvellous; it is the most wonderful tool discovered by man. No wonder Longfellow said, in Evangeline,—

> "For since the birth of time throughout all ages and nations,
> Has the craft of the smith been held in repute by the people."

There is no more remarkable development of the hammer than a modern swaging machine, like the Dayton, which is capable of delivering on one of the blanks for my little pulp-canal instruments ten thousand blows a minute, forming it into the required taper and leaving it stiff, tough, and sufficiently hard. Before leaving the description of the instruments, I mention a practice which years of experience have shown to be sound,—that of never using any pulp instrument in more than one tooth. Make the instruments at home, so their quality may be assured and their cost low. As the platinum points shown in the plate correspond in size with the drills, all that is required for filling is to cut a little from the ends so they shall not protrude, and then press them into place, covering the larger end with filling. I have found by experience that it is not necessary with fine canals to use cement or other substance about the wires, which, therefore, can be withdrawn, if necessary, for treatment of the root.

Two extracted teeth are shown in the plate. One of them illustrates the ease with which these instruments, when revolving, can follow a canal with a double curve, even going out through the end opening. The other shows a root partly cut away to prove that a considerable bend at the apex does not prevent the canal from being followed to within a short distance of the end. The fact that the instrument has passed through the apex may be indicated by a slight prick. A mark can then be placed on the shank. When withdrawn the point may be a little cut off and the wire for the filling made to correspond. I have rarely had trouble from an instrument which seemed to prick. Usually I believe there is a little life in the fragment of pulp which occupies the extreme end of the canal, and the prick comes from touching this. One can assure himself of this by an X-ray examination, for which purpose the camera I figured in this journal for 1896 is useful. Suitable tubes which give sharp pictures of small objects may be found described in my notes on X-rays in the *Electrical Review* for 1897, 1898, 1899.

## Figures and Tables

**Figure f1:**